# Medical Legislation: Victory at Last

**Published:** 1901-02

**Authors:** 


### TEXAS MEDICAL JOURNAL.

AUSTIN, TEXAS.

A MONTHLY JOURNAL OF MEDICINE AND SURGERY.

EDITED AND PUBLISHED BY

F. E. DANIEL, M. D., AND S. E. HUDSON, M. D.

Published Monthly at Austin, Texas, by Drs. Daniel and Hudson. Subscription price $1.00 a year in advance.

Eastern Representative: John Guy Monihan, St. Paul Building, 220 Broadway, New York City.

Official organ of the West Texas Medical Association, the Houston District Medical Association, the Austin District Medical Society, the Brazos Valley Medical Association, the Galveston County Medical Society, and several others.

The firm of Drs. Daniel & Hudson is dissolved, and Dr. Daniel resumes sole control and management of the JOURNAL.

### Medical Legislation: Victory at Last.

THE MEDICAL PRACTICE ACT drawn by the committee of the Texas State Medical Association—Dr. J. T. Wilson, of Sherman, chairman—passed the Senate February 19th at 5 p. m., and if the Governor signs it,—and there is little, if any, doubt that he will do so at once,—it will become a law.

The JOURNAL has great pleasure in extending congratulations to the Association and to the medical profession of Texas upon this great victory; a victory won only after twenty years of patient and persevering endeavor on the part of the profession, and by a world of labor by the various committees that from time to time have had the matter in hand. The JOURNAL rejoices with its readers and friends of humanity everywhere that at last Texas will be redeemed in a large measure from the presence of quackery and the domination, I may say, of uneducated "doctors." While the bill is not all that could be desired,—because several objectionable amendments, to be presently mentioned, had to be accepted in order to get the bill in its chief features passed through the house,—it is a big improvement on the old law, and will do a world of good.

As finally passed it provides for a *State Board of Medical Examiners* for the regular profession, and one also for each the homeopaths and the eclectics. Thus we are not to be *mixed* at all with them;—and that is the one feature of the bill for which the "Red

Back" has always contended. And because former bills provided for a mixed board—we have always opposed it, believing conscientiously that it was not for the best interests of legitimate medicine to extend to any "pathy" recognition as a "school of medicine," and that it was derogatory to the dignity of the regular profession to affiliate with them. "Their sectarian title makes them quacks," says the code, which is the pole-star of legitimate medicine. The bill *knocks out all diplomas,* and requires examination and a *license* to practice medicine. This the JOURNAL has hammered on, and insisted on, in season and out of season. The legislators have at last been aroused to an appreciation of the fact that a *diploma* is *not a license,* and should not be. Having secured these important reforms, the Association's committee and friends of the bill could well afford to concede the points fought for by the opposition,—which concession was necessary to the life of the bill;—that is to exempt from examination all those who pretend to 'heal' without drugs. That lets in the Christian science cranks, the osteopaths, *et id om. gen.* It was the best that could be done. In the next issue we will publish the law in full. We have space here and now only for this brief mention, and have stopped the press to make it. We should not fail to say, however, that the profession owes a great debt of gratitude to those by whose indefatigable labors this great reform has been secured, and we name first Chairman Wilson and his associates; and especially the representative from Dr. Wilson's county—Grayson,—Judge Walker. He made it "his fight," and secured its passage by the House. In the Senate the bill (as amended in the House) was vigorously championed by Dr. Yett, senator from Travis county district, Senator Patterson from Bell county and Senator Davidson of DeWitt. The Association will assuredly put these names high up on their roll of honor, and elect them to honorary membership. A large number of well known and able physicians, representing the best element of the profession, were in attendance upon the session of the Senate yesterday, and "got in their work" in good style. There was great rejoicing and handshaking.—Texas is now redeemed from the odium so long attached to her—as the "dumping-ground of the refuse of other states." We wanted to head this brief ed. with a rooster, but didn't have a cut so we will do the crowing ourselves.

---

A COMPULSORY VACCINATION BILL will be introduced in the House today, the outcome of a meeting of the State Association of County Physicians held recently.

---

*To Our Exchange Brethren:*

IN RE: GOULD and the defunct Philadelphia Medical Journal, which was to fill a long-felt want. "Ya-a-s," said the Texas legislator, as he placed his fingers on his mouth and knocked a fly off the hotel railing with a spurt of tobacco juice,—"Riley is a mighty good man, but he can't run a hotel." * * * If a man can't successfully run a medical journal that was so *evidently* needed to fill a long felt want, with $90,000 behind him—(enough to fill—even a rat hole)—he had better run something else—a street car, say—or run for the legislature. Goldsmith saw an acrobat leap over a pole ten feet from the ground. He said *he* could do it and skinned his shins. Gould, you all remember, didn't want any little *American* journals as exchanges—cut us all off, you know. He had a "flush" hand, *went it alone* and got *euchred.* Let up on Gould. Too much pudding will choke a dog.

